# Methodology for Enablement of Human Digital Twins for Quality Assurance in the Aerospace Manufacturing Domain

**DOI:** 10.3390/s25113362

**Published:** 2025-05-27

**Authors:** Christopher Lee Colaw, Garrett Madison, Bill Tseng, Grayson Michael Griser, Gage Truelson, Adam Gallo, Yildirim Hurmuzlu

**Affiliations:** 1 Department of Mechanical Engineering, Southern Methodist University, Dallas, TX 75205, USA; gmadison@smu.edu (G.M.); ggriser@smu.edu (G.M.G.); gtruelson@smu.edu (G.T.); galloa@smu.edu (A.G.); hurmuzlu@lyle.smu.edu (Y.H.); 2 Department of Industrial, Manufacturing and Systems Engineering, The University of Texas, El Paso, TX 79968, USA; btseng@utep.edu

**Keywords:** Human Digital Twin, virtual environment, security camera, virtual reality, artificial intelligence, detection, human feedback

## Abstract

This paper will examine a methodology to enable the usage of Human Digital Twins (HDTs) for Quality Assurance in the aerospace manufacturing domain. Common-place hardware and infrastructure, including cloud-based facility security cameras, cloud-based commercial virtual environments, a virtual reality (VR) headset, and artificial intelligence (AI) detection algorithms, have been connected via application programming interfaces (API) to enable a 24-h surveillance and feedback capability for a representative aerospace manufacturing cell. Human operators who perform defined manufacturing assembly operations in real life in the cell can utilize this methodology to digitize their performance and provide objective evidence of conformity and safety messaging for their human-centric manufacturing operation in real time. The digitization of real human-centric performance using this methodology creates the foundation for a HDT. This paper will present the application of HDTs in a manner that can easily be scaled across manufacturing operations while utilizing technologies that are already commonly inserted into existing manufacturing operations, which facilitates the exploration of HDT concepts without the need for expensive capital purchases and emerging technologies.

## 1. Introduction

Quality assurance programs in the manufacturing sector have historically been heavily dependent on human labor, which is inherently non-digital. Quality activities are classically performed by an inspector, but manufacturing personnel can also perform inspections of their own work too. Quality assessments can be focused on parts or product compliance, but can also be focused on compliance of process, such as following safety protocols. According to some studies in the manufacturing sector, as much as 80 percent of all quality assessments are made subjectively by humans based on their expertise and tradecraft [1]. Recent global trends with Industry 4.0, which sought to automate processes and eliminate humans from the equation, have shown that human presence is a critical enabler for certain types of work, such as quality assurance. The expectations, variation, and potential failure modes are vast and too complicated for full automation of all quality assurance activities. Based on this understanding, a new focus on Industry 5.0 has emerged, which aims to keep the human centered in the process and build their capability of performance through digital and technological teaming [2]. This requires a solution to digitize human performance.

HDT is a solution that can provide digital representations of human performance and is a valuable complement to the existing focus on Digital Twins of “things” that has become common under Industry 4.0 [2]. Formally defined, the HDT is a dynamic digital representation of an individual that is continuously updated to reflect and predict their physical, mental, and emotional state [3]. This representation requires the integration of data, simulation, and predictive analytics, which can be useful for the subsequent prediction of their performance of situational dependent tasks, such as quality assurance tasks in the aerospace manufacturing domain. HDTs are conceptually positioned to enable improvement of human performance and productivity, prioritize human well-being and potential, and transform the existing focus on smart manufacturing systems [4]. Like Digital Twins, HDTs are made possible through the application of sensing, analysis, and real-time feedback provided to the human, which enables personalization to the human worker while also improving the system design of the representative environment [5]. The HDT domain exhibits unique potential for identification of human stress, ergonomics, and other emotional indicators that may affect performance or safety. Biosensors and natural language processing techniques, combined with personnel skills, training certifications and behavioral assessments could be used to enable this capability in the future, while it is likely that initial progress will utilize analysis of video logs [6].

Exploration and usage of HDT concepts is still limited in the literature; however, there are preliminary applications in infrastructure construction, medical, and smart manufacturing domains. Bridge inspection has been shown to benefit from human machine collaboration, whereby the human can visualize and supervise data from independent robotic platforms based on localization techniques between the human and robots in real time [7]. In the medical domain, HDTs are used to evaluate the human condition and provide that information to the patient and relevant hospital staff [8]. In this scenario, important data are collected using smart wearable sensors and phones and relayed through the hospital network to all involved for a real update of the conditions. In both of these examples, the Digital Twin is dependent on the integration of digital and physical versions of the same twin, which is enabled by the human, sensors, machines and computer models to provide decisions and learning in the operation of a task [9]. HDT applications have been explored in Industry-4.0-based manufacturing settings with human–robot collaboration [10]. This application utilized human operators with biometric sensors and motion capture systems to analyze repetitive motion and optimize task assignments between humans and collaborative robots. There is also some exploration of using human–robot collaboration in which the human can begin to understand the intentions of their robotic counterpart [11], but this is not quite focused on the robot understanding the intentions of the human.

Considerations for HDT applications in the aerospace manufacturing domain can be learned from applications of detection and decision-making in airport security and inspection fields. It has been shown that airline security screeners need more time to develop problem-solving skills than they do for inspection or detection skills [12]; however, inexperienced screeners often exhibit human strain because they are not skilled at knowing what is important to focus on, and, as a result, they focus on problem solving more than they should. If provided with structural aid for detection performance, problem solving can be more appropriately applied. An example of such detection aid can be found in digital assistance systems for visual inspection, whereby image acquisition, defect localization, segmentation, and annotation using smart sensors and robotics, which can interface with computer-aided design models, are found to improve the inspection process [13]. Additionally, digital assistance can be provided in the manufacturing field in the form of access to influential factors that require the attention of human workers [14]. One study documented over 30 influential factors for human workers, including informational documentation, tooling, environmental factors, product features, interface and position details, and process guidance.

The sensors and technologies to excel with the HDT are not yet readily defined; however, in the manufacturing domain, there already exist tools that may form the foundation of HDT application. VR systems and security cameras are already commonplace in the manufacturing domain and can be used to capture human behavior and motion, which would enable the replication of human physical movement in a digital format [15]. Security cameras provide visual insight into human activity, posture, and movements, which can enable physical behavior to be modeled. VR technology utilizes motion tracking, which can translate human motion into digital environments, which replicate physical behaviors. Together these technologies enable a foundational capability that is required for the creation of HDTs that replicate human behavior in realistic environments. Once this foundation is achieved, future focus can be applied to biosensors and other approaches to complement and add robustness to the HDT system in the form of gesture recognition, safety assessments, human path movement, cognitive ergonomic assessments, and what-if analysis [16]. These additional capabilities help to create a model-based engineering approach that can be used to simulate and test the behavior of a manufacturing domain system in which humans play a large part [17].

Despite early emergent Industry 4.0 progress in some areas, aerospace manufacturing companies are still dependent on human labor for the performance of most manufacturing and quality operation tasks. Under this approach, there is much reliance and trust placed on humans to perform their job correctly. While manufacturing execution systems and manufacturing planning are effective at creating and tracking completion to defined manufacturing and quality steps, the objective evidence for these steps often is limited to a simple physical or digital stamp for a given step or operation. The stamps are documented in an Operations Card (Op Card), and each stamp includes approximately 2 kilobytes of information including the name of the human operator, the date, and the step number, but no real evidence that could be used to prove the compliance of the manufactured product. Rather, the stamp simply provides evidence that the step was completed. In cases where some form of further objective evidence is needed, the only method possible is to physically look at the product again, which is a source of extra cost and time due to the human labor associated with that effort. For these reasons, the digitization of human activity as represented through HDT is very important for quality assurance in the manufacturing domain.

## 2. Materials and Methods

**Figure 1:**
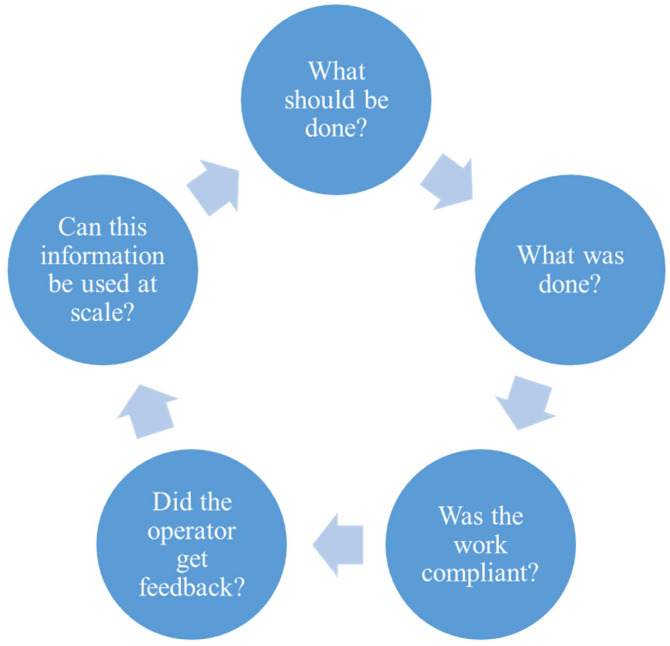
Research methodology questions to address.

Great business value could be achieved by complementing the current Op Card stamp construct with the addition of recallable and objective evidence utilizing commonplace and commercially available digital solutions. Cloud-based facility security cameras, cloud-based commercial virtual environments, VR headsets, and AI detection algorithms have emerged as commonplace capabilities by aerospace manufacturers who explored Industry 4.0. The connection of these capabilities via API can digitize and provide objective evidence of conformity and safety messaging for manufacturing operations in real time. This research methodology shall provide the capability to address the questions shown in Figure 1.

**Figure 2:**
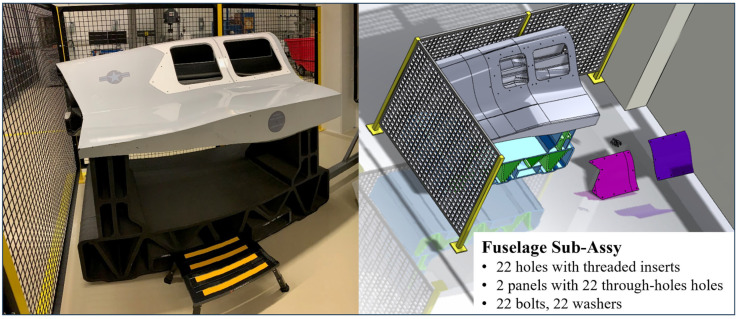
Factory work cell environment (physical and digital experimental setup).

The methodology used in this paper focuses on an aerospace representative assembly operation consisting of the placement of aircraft panels onto an aircraft body, insertion and torquing of fastening hardware, foreign object inspection of cavities, and other related in-process inspections within a factory work cell environment, as shown in Figure 2.

**Figure 3:**
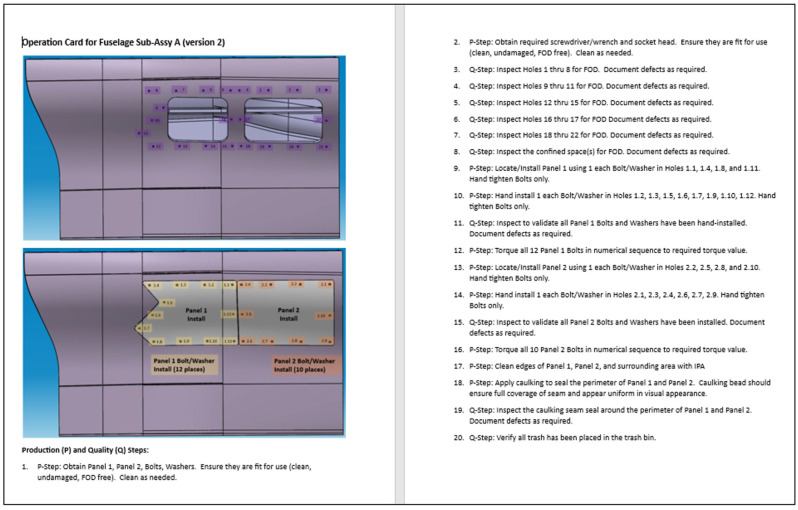
Op Card for panel assembly and inspection.

These steps are captured in the form of a manufacturing Op Card document, as shown in Figure 3, which was also translated into a VR environment to provide task guidance for the operators and capture step completions (i.e., stamps) during the performance of their task. Cloud-based facility security cameras are connected via API to the completion stamps to document objective evidence for the step and are subsequently subjected to AI detection algorithms. Variation in performance, safety messaging, and compliance status are then processed back through the virtual environment and communicated with the human operator to take applicable alternate or corrective actions. The digital capture of human performance, combined with the digital guidance of human performance, as described in this methodology, creates a foundational HDT application in the aerospace manufacturing domain.

## 3. Results

**Figure 4:**
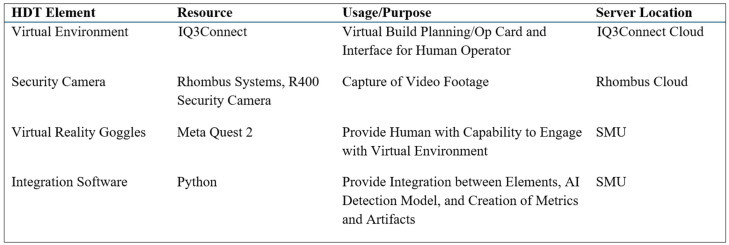
Utilized commonplace digital elements for a foundational HDT.

**Figure 5:**
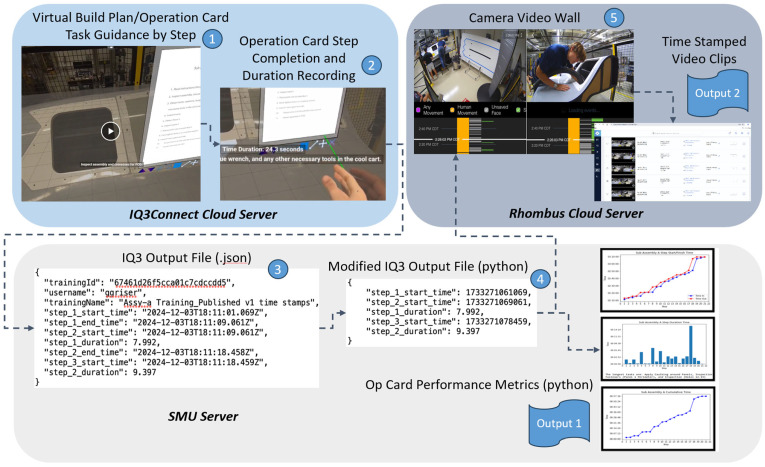
Connection of VR task guidance with security camera footage.

The cloud-based facility security cameras, cloud-based commercial virtual environments, VR headsets, and AI detection algorithms listed in Figure 4 represent independent elements across multiple server domains and must be digitally connected to create the HDT. To connect these elements, a combination of coding techniques was used to create an API approach. The first major part of this approach was focused on connecting the virtual environment, which housed the Op Card task guidance and step completion tracking, with the security camera video wall, which would be used to create video files to serve as objective evidence, and an input source for the AI detection algorithms. As shown in Figure 5, the connection process for this part includes five main process steps: (1) the virtual environment Op Card which provides task guidance to the human operator, (2) the capability to record completion, which produces a time stamp, (3) retrieval of the time stamps in a .json format from the virtual environment server, (4) conversion of the timestamps into a python input file format for the security camera server and coincidentally to produce traditional Op Card performance metrics as an output (Output 1), and (5) the combination of multiple utilized security cameras into a video wall format for real-time monitoring and creation of timestamped step-specific video files from the security camera footage (Output 2). The digital connection of these five process steps enables the creation of objective evidence that can complement the traditional Op Card stamp approach and further enable AI detection and human feedback. Please note that larger and clearer images may be found in the Appendix A.

**Figure 6:**
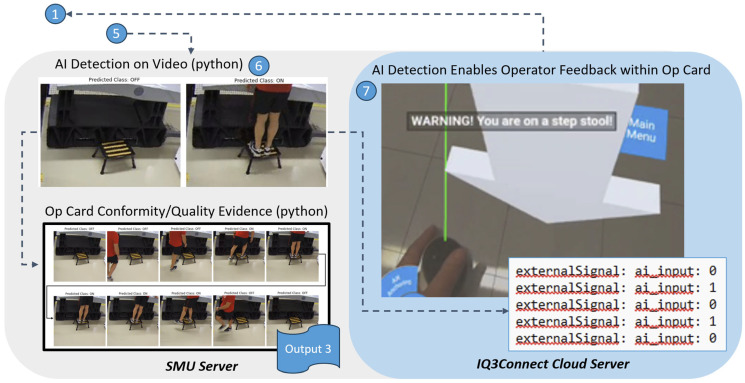
Connection of security camera footage to AI detection and a human operator.

The second major part of the HDT approach is shown in Figure 6 and is focused on usage of the security camera video wall from Step 5 as input for AI detection algorithms which are Step 6. The ultimate quantity and requisite complexity of AI algorithms for real manufacturing operations is vast; however, the foundational HDT approach can be simplified with a focus on detection algorithms to provide initial objective evidence or safety messaging for initial conditions of interest. The meta-data and image records from the detection algorithm, along with the timestamped sequence of detections, creates a new important output (Output 3), which is a valuable artifact of evidence supporting the Op Card completions. AI detection is subsequently pushed into the original virtual environment via status indicators (0 = no detection, 1 = detection), which subsequently pushes human feedback messages to the operator performing the work shown in Step 7. The modified virtual environment then becomes the new baseline for future performance and closes the loop back to Step 1 of the process. For the example shown in Figure 6, usage of the elevated step ladder is detected by the AI detection algorithm and then a warning message is sent to the operator so that they may maintain full awareness and focus on safety while performing their work.

## 4. Discussion

By way of using an initial digital environment and Op Card to direct human work, which was subsequently captured and analyzed using AI, and then communicated back to the human, a foundational HDT is formed. The new digital connections and artifacts captured in this process create greater assurance of conformity and improved safety over what is possible with traditional Op Card stamps, which essentially just document that a step was completed but provide no real evidence. These results provide a new contribution to the literature, as it was previously stated that HDT for quality assurance in the aerospace manufacturing domain is currently undocumented. While Figure 4 lists the specific resources utilized in this study, it is expected that alternative resources could also be used to deliver a HDT with the same insights. Part of the value expressed in answering the questions in Figure 1 is that the HDT is not dependent on fixed hardware and software resources. This provides value for the manufacturing industry to use the commonplace resources that they already have.

**Figure 7:**
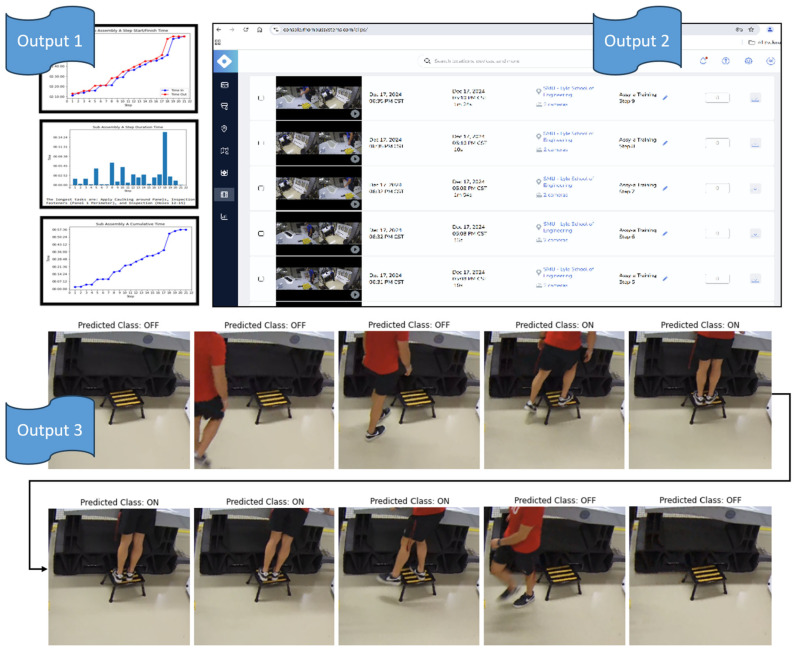
HDT digital evidence (Output 1, Output 2, and Output 3).

The HDT successfully provided artifacts and evidence, as shown in Figure 7, to address the research methodology questions asked in Figure 1. Output 1 provides a new focus on the completion of steps from the perspective of the start and stop times, which enables metrics focused on the duration of each specific step, cumulative performance time, and identification of process bottlenecks. For example, it was observed that the Op Card took 57.36 min to complete and that the longest task was Step 18, which involved applying caulking around the installed panels, which took over 14 min to complete. This approach provides manufacturing companies with wisdom and insight to drive performance improvements, unlike what that observed today, where the process bottlenecks are more subjectively felt. Regarding Output 2, the creation of timestamped video clips provides full objective evidence of what the human did during the specific Op Card step. This new evidence supports auditing, investigation, and can be used as training data for future AI algorithms. Output 3 includes the digital record of detections and associated images, which provides extreme value in the form of objective evidence for AI decision-making and overall conformity or safety of the Op Card. Subjective claims that work was performed correctly can now be proven with images and detections and can eliminate the need to reinspect, which saves money in the form of reduced human labor cost. This new evidence can also transform surveillance practices, such as safety oversight, from in-person to remote surveillance, which can save money and increase the frequency of surveillance performance. For example, it was observed by reviewing the AI detection images that the operator shown in Output 3 is leaning approximately 30 degrees while standing on the step ladder, which could be a concern to address from a safety perspective. Observation of this leaning condition would only traditionally be found through human observation but can now be detected through the review of the AI detection images. In fact, new AI algorithms, which can measure leg and trunk angles and provide further messaging to the human, may be inserted into the process.

Another important result to document is the latency in the system of providing feedback to the operator. Regarding Figure 4, near-zero latency was observed in Steps 1 through 5 and Output 1; however, the process of obtaining video clips as Output 2 was found to take up to approximately two hours in some cases. The delay was due to variation in cloud-based upload of the video stream combined with Wi-Fi signal-strength-dependent performance on the download of the video clips. It became clear that these clips, while good for objective evidence, could render the HDT solution useless if they were the subject of subsequent AI applications because the waiting time would exceed the entire Op Card duration. As an alternative to this delay, the approach to AI focused instead on usage of the raw video stream from the video wall. In this construct, the AI would log into the video stream and screen-capture raw video at a given frequency. These images were then passed through the detection algorithm in Step 6. The raw stream operates at approximately a 10-s delay from real time, which therefore inserts an overall latency of 10 s into Step 6, which is only minimally impactful to the operation being performed. After AI detection, Step 7 occurs with no additional delay; however, because of the 10-s delay in Step 6, there is a perceived delay in the receipt of the human feedback.

Finally, as with all digital systems, there is an initial setup time of approximately 5 min to turn on digital devices, open applications, address two-factor authentication requirements for IQ3Connect and Rhombus, and to align the virtual experience with the VR headset. Once this initial setup is complete, any additional performance of the same Op Card could be performed without any additional setup time. Changes to other Op Cards or manufacturing settings would require a similar, albeit less of, setup time because the devices are already turned on.

## 5. Conclusions

This paper presented a methodology to enable the usage of HDT for quality assurance in the aerospace manufacturing domain. Commonplace hardware and infrastructure, including cloud-based facility security cameras, cloud-based commercial virtual environments, a VR headset, and AI detection algorithms have been connected via API to create a capability to digitize and provide objective evidence of conformity and safety messaging for human-centric manufacturing operation in real time. The digitization of human-centric performance using this methodology creates the foundation for an HDT which can easily be scaled across manufacturing operations because the utilization of existing commonplace solutions facilitates the exploration of HDT concepts without the need for expensive capital purchases and emerging technologies. As the manufacturing sector builds experience with HDT solutions, future research into incorporation of additional sensors and inputs can be carried out and build upon the foundation laid in this paper.

## Figures and Tables

**Figure A1:**
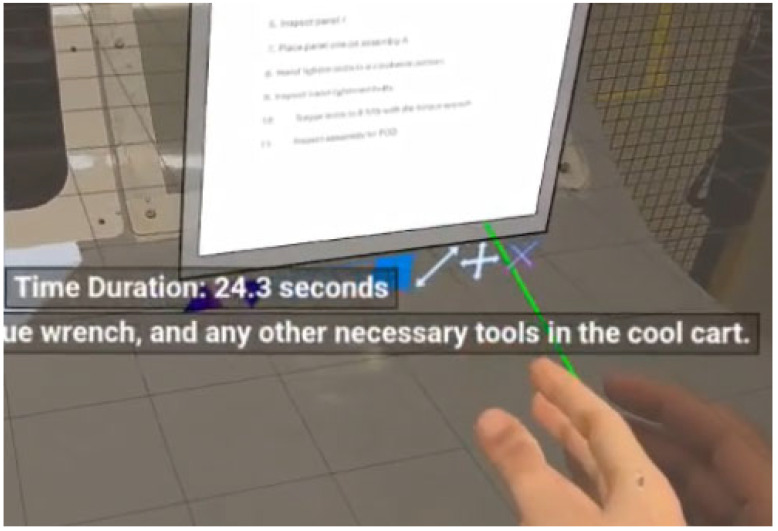
Operation Card Step Completion and Time Duration Recording (Figure 5, Process Step 2).

**Figure A2:**
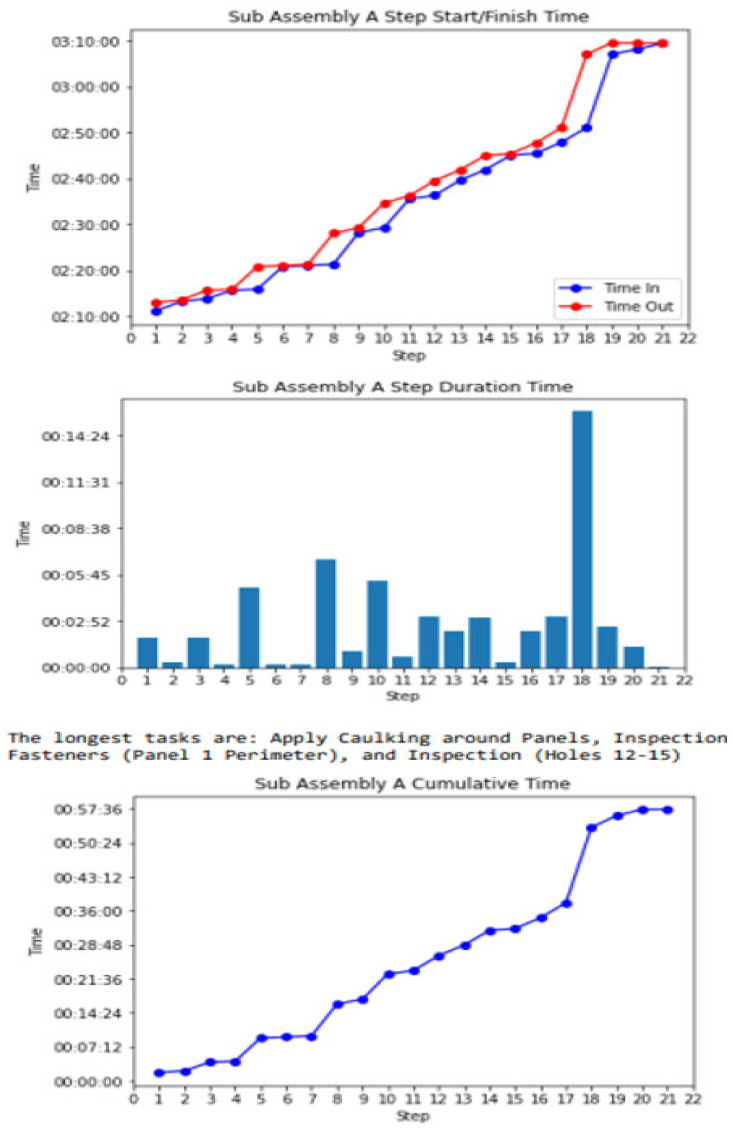
Op Card Performance Metrics (Figure 5, Output 1).

**Figure A3:**
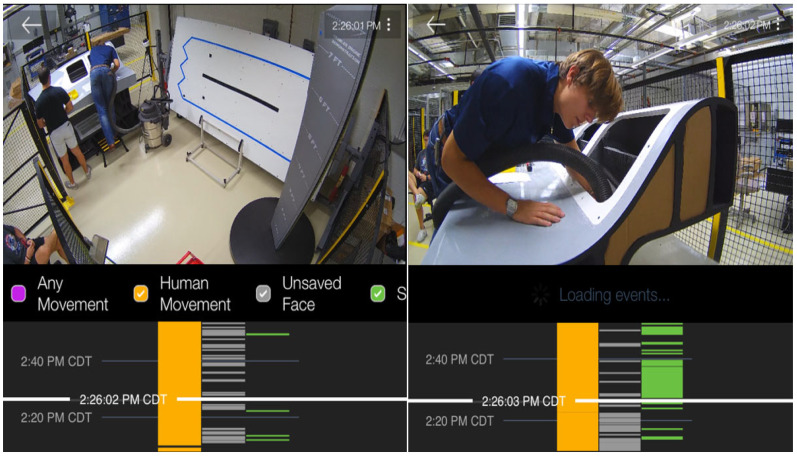
Camera Video Wall (Figure 5, Process Step 5).

**Figure A4:**
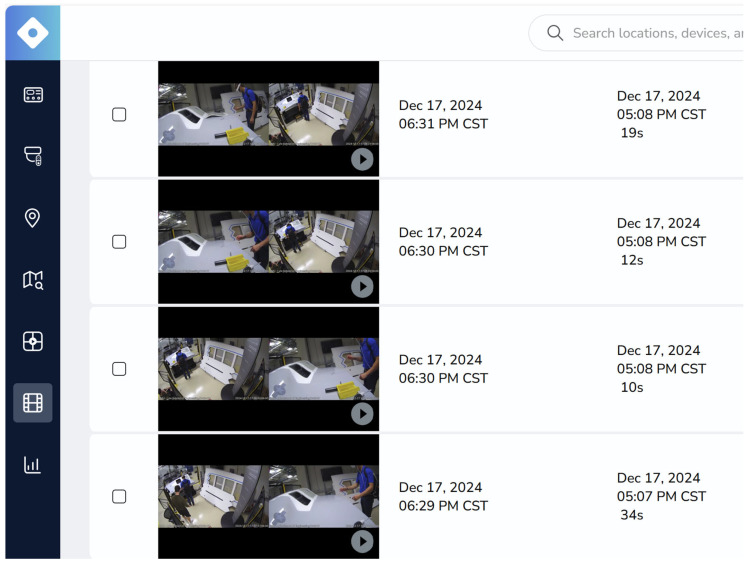
Example of Video Clip Creation from Completed Steps (Figure 5, Output 2).

## Data Availability

No publicly archived datasets were used in this study.

## Abbreviations

The following abbreviations are used in this manuscript:

HDT	Human Digital Twin
API	Application Programming Interface
VR	Virtual Reality
AI	Artificial Intelligence
Op Card	Operations Card
SMU	Southern Methodist University
CDHAM	Center for Digital and Human-Augmented Manufacturing
UTEP	University of Texas at El Paso

## Appendix A

This appendix presents larger sized images that were utilized in Figure 5. These larger images are provided for further detail and context regarding the Results.
